# From Consumers to Producers: Three Phases in the Research Journey With Undergraduates at a Regional University

**DOI:** 10.3389/fpsyg.2018.02770

**Published:** 2019-01-17

**Authors:** Ranjana Dutta, Travis J. Pashak, Jennifer D. McCullough, Joseph S. Weaver, Michael R. Heron

**Affiliations:** ^1^Department of Psychology, Saginaw Valley State University, University Center, MI, United States; ^2^Department of Communication, Saginaw Valley State University, University Center, MI, United States; ^3^Department of Social Work and Youth Services, Saginaw Valley State University, University Center, MI, United States

**Keywords:** publish with undergraduates, mentoring undergraduates, research at regional university, faculty mentors for undergraduate research, psychology and social sciences

*As the daughter of a single mom, I took care of my younger siblings and worked two jobs to keep the family afloat. I knew I HAD to attend college if I wanted a different life. Talking to the faculty members at orientation made me decide to come here*^1^*.—Student 1*

My advisor kept me involved in her project. She had faith in me, and that kept me motivated despite working full-time and commuting from out of town! We presented at a conference and later published it in a journal!—Student 2

## Introduction: Unique Characteristics of Regional Institutions

**Figure 1:**
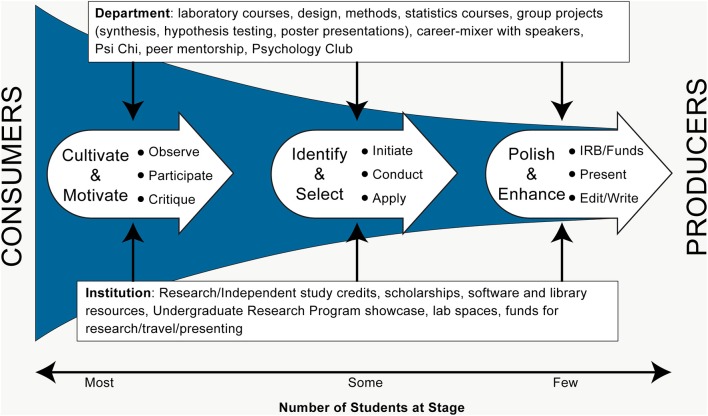
A framework for the faculty-facilitated journey of undergraduates from consumers to producers of research.

In this brief paper, we articulate suggestions and best practices for social science faculty to successfully mentor publishable research with undergraduate students. Valued for shaping students' career-choices across fields (Lent et al., 1994; Robnett et al., 2015; Frantz et al., 2017), we argue that regional universities present unique considerations which can complicate the process. Recent data show our student body is comprised of 33% first-generation college students—which often means they arrive less familiar with the cultural milieu of higher education, less prepared for the level of critical thinking required, and with less value of scholarly creation. Further, many commute, have transferred from community colleges, and work substantial hours to afford college. These characteristics mean that our students juggle numerous time-demands and view academics through a lens of efficiency (i.e., aiming to complete necessary tasks as quickly as possible to progress from college to career). They often view course content as static knowledge to be received and regurgitated. Per APA guidelines (American Psychological Association, 2013), we argue that the best undergraduate outcomes include augmenting that mindset from *consuming* empirical knowledge to critically examining and eventually *producing* scholarly information. In this paper, we outline three phases of this journey with students—cultivating/motivating, identifying/selecting, and enhancing/polishing skillsets to produce research (see Figure 1).

## Phase 1: Cultivate and Motivate

Many of our incoming students—are unfamiliar with the details, design, and value of empirical studies, so they struggle with consuming (let alone producing) research. Thus, the first phase is to kindle students' interest in the process of knowledge creation. Whether discussing how some research shaped existing views, demonstrating experiments with counterintuitive results, or doing analysis of research articles, a motivated teacher excites students about research and makes it relatable. Class-discussions repeatedly raise the question of “how we *know*?” We develop student competencies and increase self-efficacy via research-participation-, constructive evaluation of peers' research, critical analysis of current events through the lens of research methodology, and brainstorming new ways to replicate/modify studies (Hurtado et al., 2009).

The success of this phase rests on the planned pervasiveness of these tasks across the departmental and institutional curriculum so that students are consistently motivated toward research. Curricula should be designed to include research skills to produce knowledge in all students (Chamely-Wiik et al., 2014). The Association of American Colleges and Universities' VALUE rubrics (Association of American Colleges and Universities, 2009) offers recommendations. The Psychology department fosters research through various lab courses and a departmental poster session each semester to showcase student research projects. Faculty judiciously plan to involve first year students to participate in these projects for extra-credit and later attend the poster session to see the outcomes. We also deliberately plan a concurrent Psi Chi induction ceremony to model the value of research and build self-efficacy in junior students. Additionally, to motivate students, we offer “Career Preparation” mixers where invited guests provide experience-based insights into graduate school applications. At these informal mixers even the most diffident students can feel free to network with the faculty and the guest speakers and gain relevant career information. With less savvy students, we need to advise students on how to pull together institutional resources (such as specific grants for independent/faculty-mentored research). We now organize an annual, university-wide, undergraduate research showcase to create an echo-chamber to cultivate research skills and motivate students.

## Phase II: Identify and Select

This phase involves identifying and providing opportunities to students who demonstrate potential for growth and success. At regional universities, many students lack readiness (e.g., deep-reading, argumentative writing, analytical thinking, interpersonal confidence, seeking mentoring, professional networking) which confound their initial appearance of competence. Thus, the faculty's task of detecting student potential is crucial and entails opening doors to those who are diamonds in the rough (inquisitive, skeptical, and insightful) and give them opportunity to show their talent with helpful mentorship in addition to those who are conspicuous or have good grades. This search for research acumen diversifies future researchers and allows us to live our mission as regional university faculty: providing *opportunity* to those less privileged. Indeed, mentored research facilitates frequent student-faculty contact, enhanced self-efficacy, and bolstered science identity, which in turn improve college experiences for underrepresented minorities and may help shrink the disparities between racial groups such as graduation rates and admission rates to graduate schools (Hurtado et al., 2009).

Identifying potential takes many forms and each faculty member handles this slightly differently providing opportunity in addition to the objective criteria of selection on grades and skills. Some commonalities we suggest are to look for potential in creative, skeptical, open-minded, detail-oriented students; market opportunities widely and include the hesitant along with the self-promoting students; consider team composition and synergy between students; include and pair promising students with experienced students to scaffold learning and assess growth with mentorship; seek out and encourage underrepresented student groups; encourage faculty mentors with resources; offer course credit, scholarships, or payment to student researchers so they do not have to choose between research and financial stability; and lastly, implement a formal application process. A formal process is doubly helpful because the applicant's CV/transcripts/essays serve as data for making wise choices to supplement interpersonal assessments, and it socializes students on the significance and preparation of these materials ahead of their eventual applications to jobs and/or graduate/professional schools.

To be successful, the process of identification and providing opportunity needs to be baked into the departmental and institutional culture. Collegial relationships benefit students when faculty members refer them to their colleagues with matching research interests. Thus creating otherwise missed mentorship opportunities. Further, the institution-wide dissemination opportunities for student research improve visibility of students, help us network across departments, build awareness of up-and-coming research programs, and spark novel research ideas. Each of these opportunities connect students to one another and creates an ethos that nurtures the production of scholarship, while also connecting faculty and university administrators. To other research mentors at regional universities, we strongly recommend taking a similar approach to identifying and providing opportunity to future mentees on their potential in addition to observed competence.

## Phase III: Polish and Enhance

The final stages of producing with students (including posters and papers at conferences at the university, regional, or national level) can stall due to obstacles. As most research students are seniors, their availability and timelines are often not congruent with the length of time it takes to publish. For the challenges associated with this final stage of the journey, we offer added suggestions.

To enhance and polish research skills we have found the most success by delicately balancing “hands on” techniques with delegating meaningful tasks to pursue independently. We enhance skills by emphasizing, modeling, and adding important details—(logs of procedures, making analytic decisions explicit, suitability of measures, programming of stimuli, replicability of procedures and results). Besides orchestrating the pairing of novice and skilled peers to polish and enhance specific skills (such as analysis or writing), we also directly intervene. For example, we polish their communication skills in synthesizing research by giving independence to try tasks followed by immediate and collaborative feedback, such as coediting a manuscript or speech and including them on professional correspondence. We enhance career skills by encouraging students to create an online presence and follow communication etiquette with professionals in the field (e.g., LinkedIn, ResearchGate, Mendeley). Presenting at regional or national research conferences is always an eye-opening experience and makes tangible the significance of clear dialogue in a research community (Gumbhir, 2014). To aid this we help them navigate conferences with us and overcome inhibition to speak with colleagues. We even examine journal outlets with them and rewrite drafts to hone the argument for the paper to fit with the literature giving them glimpses into our own incessant learning. Lastly, we encourage conversations about mutual expectations of interpersonal interactions to co-create meaningful mentor-mentee relationships (Shanahan et al., 2015).

Encouraging them to disseminate the knowledge produced, we conjointly examine conferences, peer-reviewed journals, and look for other creative ways of publishing which fit with the limited budgets of time (given heavy teaching loads) and funding for undergraduate research. We keep an eye for respectable non-peer-reviewed outlets as well (e.g., books, magazines, Twitter, blogs), as exemplified by a faculty member's ambitious project of coaching his entire class to co-author a book in social psychology (Fairchild and Fairchild, 2018). We are encouraged by non-traditional outlets such as the Wikipedia initiative of the Association for Psychological Science (APS), encouraging teachers to build writing skills with students by contributing to Wiki pages (Banaji, 2011). Such projects could serve as scaffolds of writing experiences for students on the way to professional dissemination in peer-reviewed journals.

Institutional policies and procedures are pivotal to creating the culture of research which is facilitative or prohibitive of the final steps on the students' journey from consumer to producer (Brew and Mantai, 2017). Institutions enhance the likelihood of publications by committing resources to funding the dissemination of research. Our institution supports polishing and dissemination of findings through a writing center where students can get feedback, a university-wide research symposium, financial support for conference presentations, and as well as institutional initiatives to publicize and promote awareness about colleagues participating in conferences or publishing through campus-wide newsletters, stories on websites, and press releases. The departmental poster sessions and the University showcase are the perfect low-risk environments for students to further polish their skills as they get feedback on design, analysis, and presentations from peers and other faculty members as well. At a regional university, this interconnecting of disparate functions which is largely done by committed faculty allows students (and the mentoring faculty) to consult with other expert faculty on methods, statistics, stimuli, questionnaire design, literature, and manuscript production.

## Conclusions

Although the strategies suggested in this paper would be beneficial at any university, we believe they are particularly critical at a regional university. The role of faculty in bringing together departmental and institutional resources is uniquely pivotal and takes personal investment to help undergraduates navigate their journey from consumers to producers. Unlike other institutions, we engage with students from the outset, often even before they are admitted. Not only do we mentor students, we build their competence, cheer their accomplishments, and bolster their confidence all through their journey with us, and support them even after they graduate. We pay forward to the next generation of culturally diverse, economically challenged, and often first-generation students that come to us. Encouraging mentors of undergraduate research with funds and ongoing training is a great investment, as facilitating such research activities in social sciences at regional universities may be an important path to diversify future scientists and creators of knowledge (Meadon and Spurrett, 2010).

*Seeing my name on a published paper was pretty crazy… like “I DID THAT!” The research process really helped me understand where knowledge comes from and helped me to see myself as having the potential to contribute to this field.—Student 3*.

## Author Contributions

RD developed the initial ideas and submitted abstract along with TP as coauthor who also publishes with undergraduates and added additional ideas of how he manages to research teams and publication process. JM, JW, and MH added their thoughts on how they mentor research in fields of Communications and Social-Work. JW also assisted with making the figure. Additionally, JM provided the institutional perspective as she is Director of Undergraduate Research at the University.

### Conflict of Interest Statement

The authors declare that the research was conducted in the absence of any commercial or financial relationships that could be construed as a potential conflict of interest.

## Footnotes

^1^The quotes are fictive, representing synthesized conversations, and not a particular student.
